# Growth and Primary Metabolism of Lettuce Seedlings (*Lactuca sativa* L.) Are Promoted by an Innovative Iron-Based Fenton-Composted Amendment

**DOI:** 10.3390/plants12122234

**Published:** 2023-06-07

**Authors:** Amalia Piro, Daniela Oliva, Dante Matteo Nisticò, Ilaria Lania, Maria Rita Basile, Giuseppe Chidichimo, Silvia Mazzuca

**Affiliations:** 1 Laboratory of Plant Biology and Plant Proteomics (Lab.Bio.Pro.Ve.), Department of Chemistry and Chemical Technologies, Università della Calabria, 87036 Rende, Italy; daniela.oliva@unical.it (D.O.); dante.nistico@unical.it (D.M.N.); silvia.mazzuca@unical.it (S.M.); 2 Physical Chemistry (CFINABEC) Laboratory, Department of Chemistry and Chemical Technologies, Università della Calabria, 87036 Rende, Italy; ilarialania.il@gmail.com (I.L.); basimari88@gmail.com (M.R.B.); giuseppe.chidichimo@unical.it (G.C.)

**Keywords:** food waste compost, Fenton reaction, plant growth, plant proteomics, *Lactuca sativa* L., content in chlorophyll

## Abstract

Information regarding the physiological and molecular plant responses to the treatment with new biofertilizers is limited. In this study, a fast-composting soil amendment obtained from solid waste by means of a Fenton reaction was assessed to evaluate the effects on the growth of *Lactuca sativa* L. var. longifolia seedlings. Growth rate, root biomass, chlorophyll concentration, and total soluble proteins of seedlings treated with the 2% fast-composting soil amendment showed significant increases in comparison with the control seedlings. Proteomic analysis revealed that the soil amendment induced the up-regulation of proteins belonging to photosynthesis machinery, carbohydrate metabolism, and promoted energy metabolism. Root proteomics indicated that the fast-composting soil amendment strongly induced the organs morphogenesis and development; root cap development, lateral root formation, and post-embryonic root morphogenesis were the main biological processes enriched by the treatment. Overall, our data suggest that the addition of the fast-composting soil amendment formulation to the base soils might ameliorate plant growth by inducing carbohydrate primary metabolism and the differentiation of a robust root system.

## 1. Introduction

In recent decades, there has been a strong demand to gradually replace intensive agriculture with sustainable cultivation practices, which are based on the use of organic amendments that are able to preserve the integrity of the soil and the healthiness of crops [1,2,3]. Plants need not only light and water for their metabolism, but also a complete mineral nutrition, whose availability is governed by soil properties and the efficiency of water uptake by the roots. Currently, the functionality and productivity of the soil are seriously compromised by the intensive use for crops and by the degradation of fertile soils [4]; the use of biofertilizers in agriculture help plants to enhance their absorption of nutrient elements from the soil and improve their growth [5]. The production of organic fertilizers from the urban organic waste is expected to increase, and the challenge at the moment is to tackle these problems together by transforming food waste into a sustainable resource to enrich the soils and enhance their nutritional quality and regeneration. Among the waste sources, those of the food residues and the municipal biosolids seem to be the most promising in the urban sustainable recycling process [6]. Unfortunately, current recycling processes take months to degrade organic wastes, thus representing the main limiting factor for a large-scale application. Alternative processes, based on waste drying or high-temperature aerobic fermentation were advanced to overcome this limitation [7,8]. Nowadays, the classical Fenton reaction [9] is considered as one of a set of advanced oxidation processes (AOPs) and is widely used for the chemical treatment of wastewater, industrial sludge, landfill leachate, soils, and sediments, which are contaminated with biorefractory organic compounds including phenols, dyes, pesticides, organic solvents, pharmaceuticals, and domestic chemicals [10]. Specifically, the fast degradation of urban solid waste using the Fenton reaction has already been achieved [11]; the authors reported that after a few hours of incubation, the Fe (II) ion catalytic oxidation pathway was able to degrade dry organic matrices with a C/N ratio lower than 12, with a high degree of oxidative decomposition converted into low-molecular-weight compounds at a high-oxidation state.

The aim of this research was to assess the effects of the soil amendment formulation reported as the fast-composted soil amendment (FCA), obtained by urban, organic solid waste fractions following the Fenton composting reaction on the growth of *Lactuca sativa* L. seedlings. Lettuce is one of the most intensively cultivated vegetables in Mediterranean countries. Millions of tons of lettuce for fresh consumption are produced annually in the EU, mainly in Mediterranean countries (FAO, 2022). Intensive lettuce cultivation practices are large consumers of fertilizers, thus entailing significant costs in terms of energy and the environment. Therefore, providing alternatives that improve the sustainability of these agricultural ecosystems without reducing productivity is of great interest. Under our experimental conditions, *L. sativa* was grown in a short-day period to prevent flowering and thus evaluates plant growth in its vegetative phase. Morphological, biochemical, and molecular analyzes have been performed to elucidate the metabolic behavior of the leaves and roots of plants grown on soil enriched with the FCA.

## 2. Results

### 2.1. Morphological and Biochemical Behavior of Seedlings Treated with Fast-Composted Soil Amendment (FCA)

**Figure 1:**
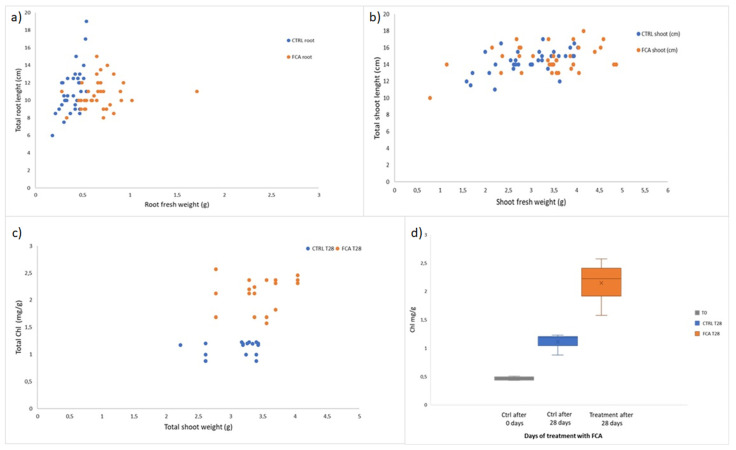
Morphological and biochemical parameters measured in seedlings *Lactuca sativa* after 28 days cultivation. Qualitative responses for each parameter in the treated samples with the iron-based Fenton-composted amendment (FCA) with respect to the controls (CTRL) have been reported. Dot plots of (**a**) root fresh weight vs. total root length ratio; (**b**) shoot fresh weight vs. total shoot length ratio; (**c**) total shoot weight vs. total chlorophyll content ratio; and (**d**) box plots of total chlorophyll content at the initial day of treatment (T0) and after 28 days in the CRTL and FCA samples. Standard deviation (SD) and student’s t-test have been calculated for significance with values lower than *p* < 0.05. (**a**,**b**) *n* = 35 each treatment; (**c**,**d**) *n* = 18 each treatment.

In Figure 1, the morphological and biochemical parameters of the *L. sativa* seedlings grown on the control soil and FCA-added soil for 28 days have been reported. As a consequence of the FCA treatment, roots showed significant increases mainly in the weight rather than in the total length (Figure 1a), while the shoots length and weight were not significantly affected by the treatment (Figure 1b); total chlorophyll concentration significantly increased after 28 days in the FCA-treated shoots with respect to the control samples (Figure 1c). During the cultivation period, the total chlorophyll content in the control shoots also raised and reached a two-fold increase after 28 days in comparison to the chlorophyll content at day 0 (T0), while in FCA-treated shoots, the total chlorophyll content was found to have increased by four-fold in comparison with value at T0 (Figure 1d).

### 2.2. Quantitative Analysis of Leaf and Root Proteins of Lactuca sativa Seedlings Treated with FCA

#### 2.2.1. GO Enrichment of Biological Processes in *L. sativa* Leaves and Roots Treated with FCA

**Figure 2:**
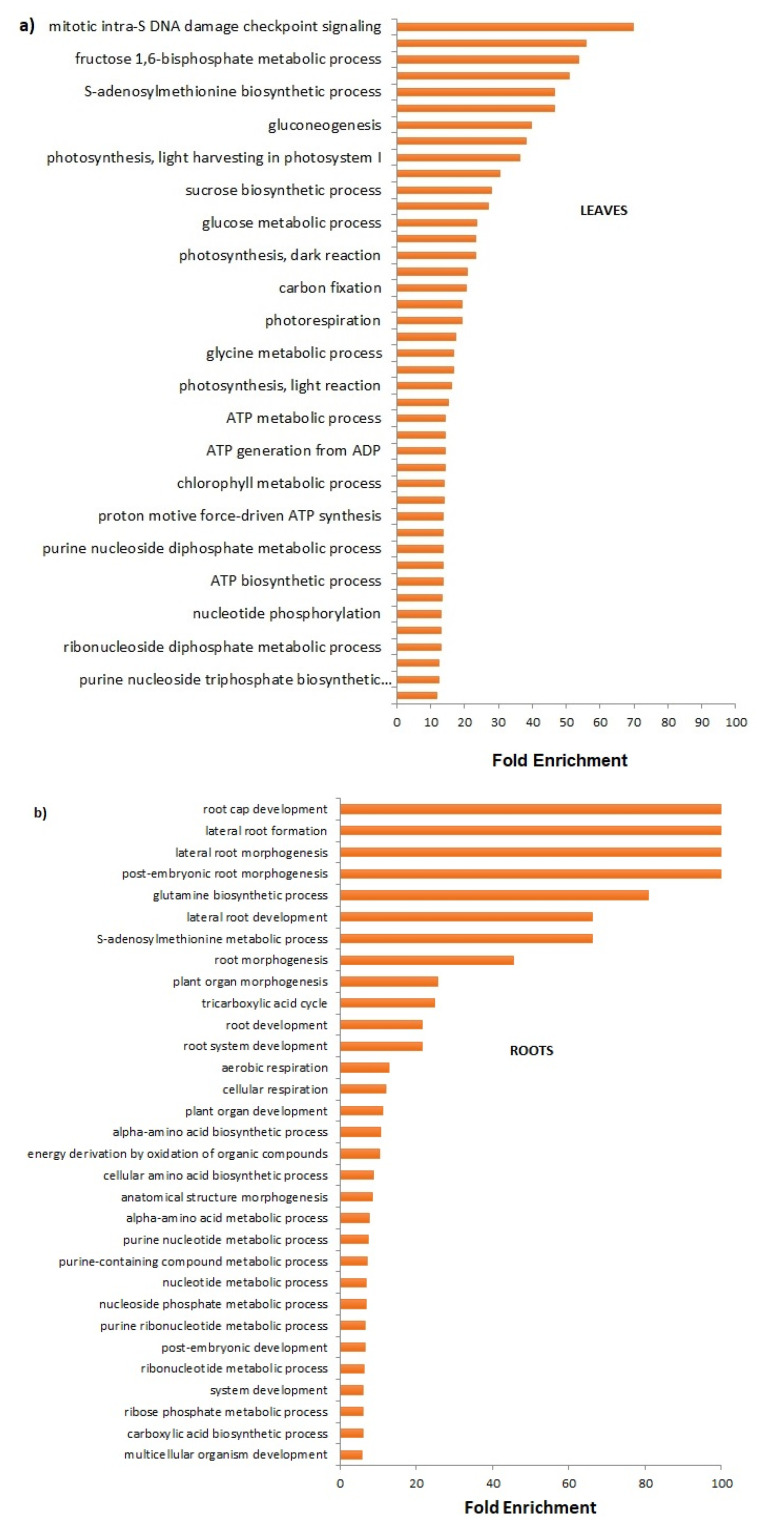
Sequence distribution from GO-enriched terms analysis for biological processes of differentially accumulated proteins in the leaf (**a**) and root tissues (**b**) of *L. sativa* seedlings treated with FCA.

Quantitative analysis revealed that 401 proteins varied their expression pattern in the leaves and 192 in the roots following FCA treatment (Supplementary Tables S4 and S5, respectively). The GO assignments and GO enrichment of biological processes in the treated leaves and roots of all differentially accumulated proteins (DAPs) were shown in Figure 2. The fold enrichment for each biological process was obtained from the ratio between the expected and in the sample number of sequences found (Supplementary Tables S4 and S5, GO enrichment). Among the widely enriched biological processes, the mitotic DNA damage response signaling, the S-adenosylmethionine biosynthetic process, glycolysis, photosynthesis light and dark reactions, carbon fixation, photosynthetic electron transport in photosystem II, photorespiration, carbohydrate biosynthesis, and glycine metabolism were the most represented in the FCA-treated leaves (Figure 2a). In roots, the treatment with FCA mainly induced the enrichment of biological processes related to root cap development, lateral root formation, and morphogenesis, along with glutamine biosynthesis and S-adenosylmethionine metabolism (Figure 2b). Both the raw *p*-values and FDR-corrected values are reported in the Supplementary Tables S4 and S5.

#### 2.2.2. Distribution Histograms of Expression Pattern in Leaves and Roots

**Figure 3:**
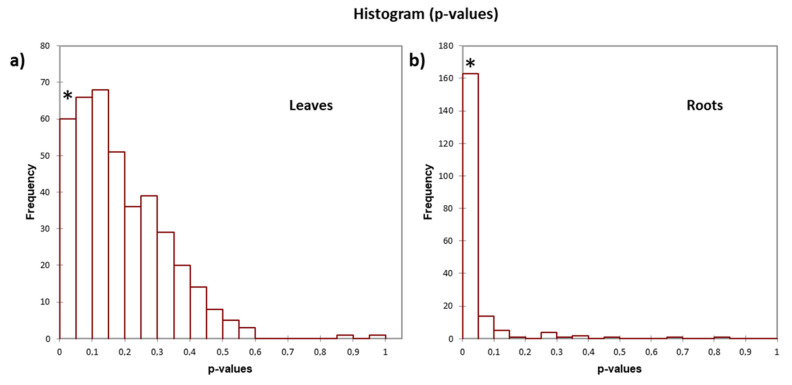
Distribution histograms in the range of p-values of protein that varied their expression pattern in the (**a**) leaves and (**b**) roots of *L. sativa* seedlings treated with FCA compared to control plants. Details of the analyses are shown in the Supplementary Tables S1 and S2. * 0.0001 ≤ *p* ≤ 0.05 (XLSTAT 2022.6.1.1187—Differential expression tool). In roots, 59 proteins were significantly depleted (−0.5 < LogFC < −1.8; *p* < 0.001) and 103 were accumulated (0.2 < LogFC < 2.6; *p* < 0.001).

Among all DAPs, only 60 proteins exhibited variation patterns with a high significance among leaf samples, while in root samples, a greater number of proteins (163) had a significant variation (Figure 3a,b, see stars). Differential abundance analysis of the proteins quantified in the FCA-treated and control seedlings are also depicted as volcano plots in the leaves (Figure 3a) and in the roots (Figure 3b); red spots appearing on the left side of the graphs are proteins whose quantities decreased as a result of the treatment, while the red spots on the right side are those accumulated by the treatment; 40 proteins were significantly depleted (−0.5 < LogFC < −2; *p* < 0.001) and 18 accumulated (0.5 < LogFC < 3; *p* < 0.001) in leaf tissues following the treatment with FCA (Figure 2a, spots up to the dotted line).

#### 2.2.3. Differentially Accumulated Proteins (DAPs) Identified in *L. sativa* Roots Treated with FCA Amendment

**Figure 4:**
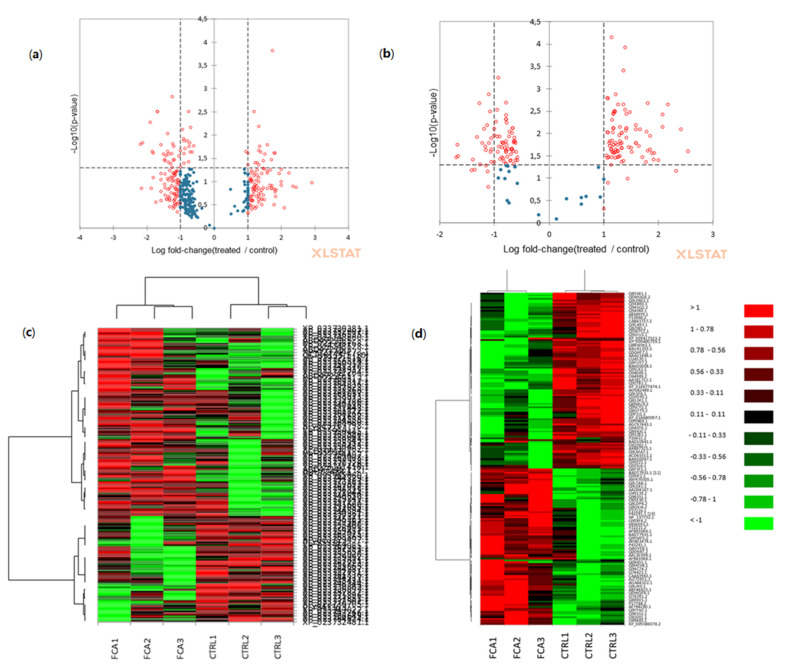
Graphical representation of differentially expressed proteins in leaves treated with FCA compared to the control. Volcano plots that display and identify statistically significant changes in protein expression in FCA-treated (**a**) leaves and (**b**) roots in terms of change in the Log fold-change values (X axis) and p-values (Y axis). Heat maps of proteins differentially expressed at various sampling times in FCA and control samples in the (**c**) leaves and (**d**) roots. Protein expression values were normalized to log2, and cluster analysis was performed using the fold-change levels of proteins. The color code panels on the right indicate the described preferential expression of a protein. (XLSTAT 2022.6.1.1187—Differential expression tool). The details of the analysis are shown in Supplementary Tables S1 and S2.

Heat maps showing an unsupervised clustering of the samples and proteins based on their abundance and similarities occurring in the FCA-treated leaves and roots, compared with the controls, are presented in Figure 4c,d. Sample/proteins were clustered via correlation distance and hierarchical agglomerative clustering, and colors were scaled per row. As illustrated in the figure, among the root biological replicates, the clusters of accumulated/depleted proteins inverted their patterns significantly following the FCA treatment; in leaves, there were low significant correlations observed among the replicates, patterns, and treatment for the accumulated/depleted. 

#### 2.2.4. Functional Classification and KEGG Pathway Enrichment of DAPs in Leaves and Roots of *Lactuca sativa* Seedlings Treated with FCA

**Figure 5:**
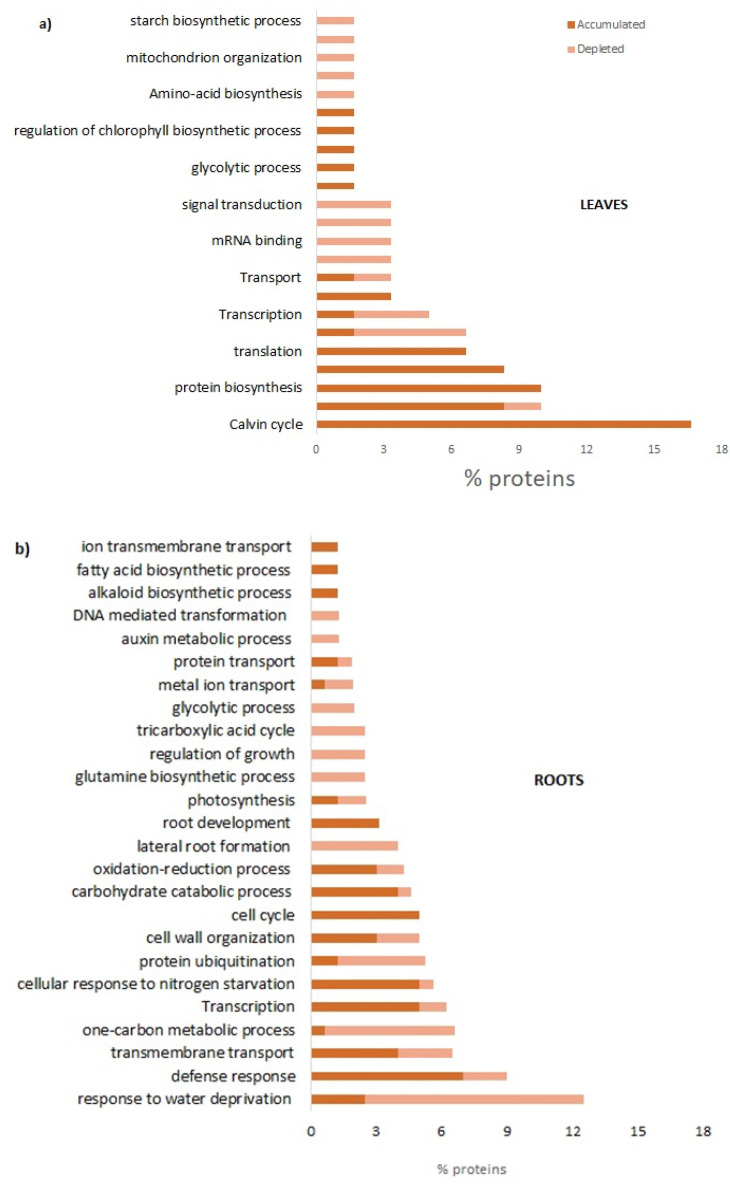
Percentages of accumulated or depleted proteins belonging to the main functional annotations that had significant variations in the leaf (**a**) and root tissues (**b**) from *L. sativa* seedlings treated with FCA.

When the functional classification (evaluated by their GO terms) of the accumulated or depleted proteins have been deciphered in the leaves, the main accumulated proteins were found to belong to the Calvin cycle; the ribulose-1,5-bisphosphate carboxylase/oxygenase large subunit corresponded to sixteen percent of DAPs; among the photosynthesis, the chlorophyll a-b binding protein of LHCII type 1, and the oxygen-evolving enhancer protein 1 were accumulated three-fold and two-fold, respectively, compared to the control leaves. The elongation factor 1-alpha 1 was found to be the most abundant protein belonging to protein biosynthesis metabolism, corresponding to ten percent of DAPs. The chloroplastic ATP synthase CF1 alpha subunit translation and the 60S ribosomal protein L4 were two-fold accumulated; the first is involved in the ATP synthesis-coupled proton transport in thylakoids, and the second is involved in protein biosynthesis. Chloroplast organization, glycolytic process response to stimulus, and proton export across the plasma membranes were also found to have accumulated. Among other functional classifications, the DNA repair, mRNA binding, oxidation-reduction process, and signal transduction were all found to have depleted (Figure 5a).

In roots, proteins belonging to the response to water deprivation corresponded to twelve percent of the total DAPs in which the aquaporin PIP-type was the main depleted protein of up to three-fold less than the control. The cysteine proteinase inhibitor and the SPX domain-containing protein 1, belonging to the defense response, were both fund to have accumulated by four-fold. Among the processes that also accumulated, SPX domain-containing protein 1, Protein PHR1, protein decreased size exclusion limit 1, and class IV chitinase were the main proteins observed. Patatin-like protein was the only protein depleted in the root development process (Supplementary Tables S4 and S5).

**Figure 6:**
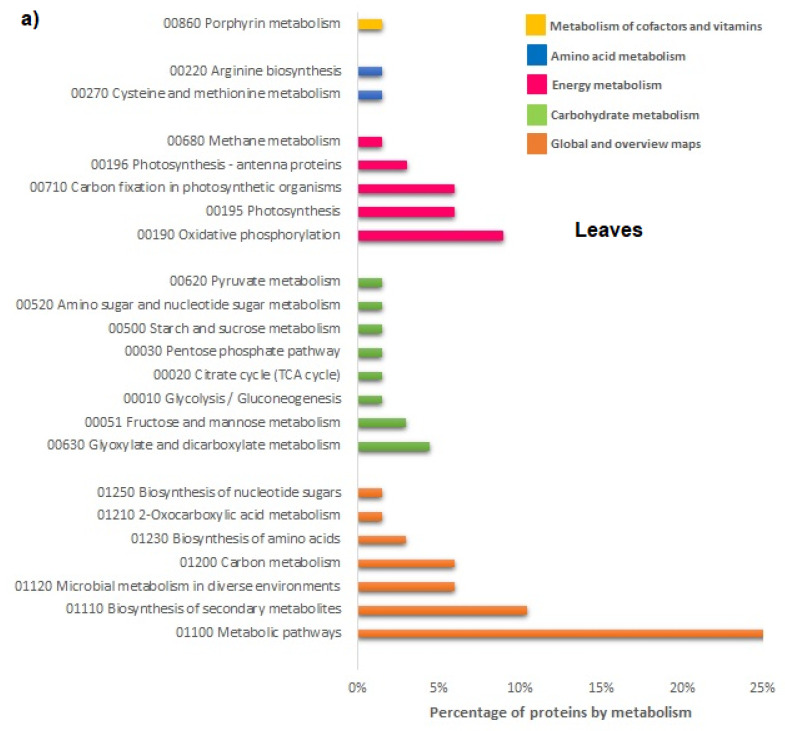

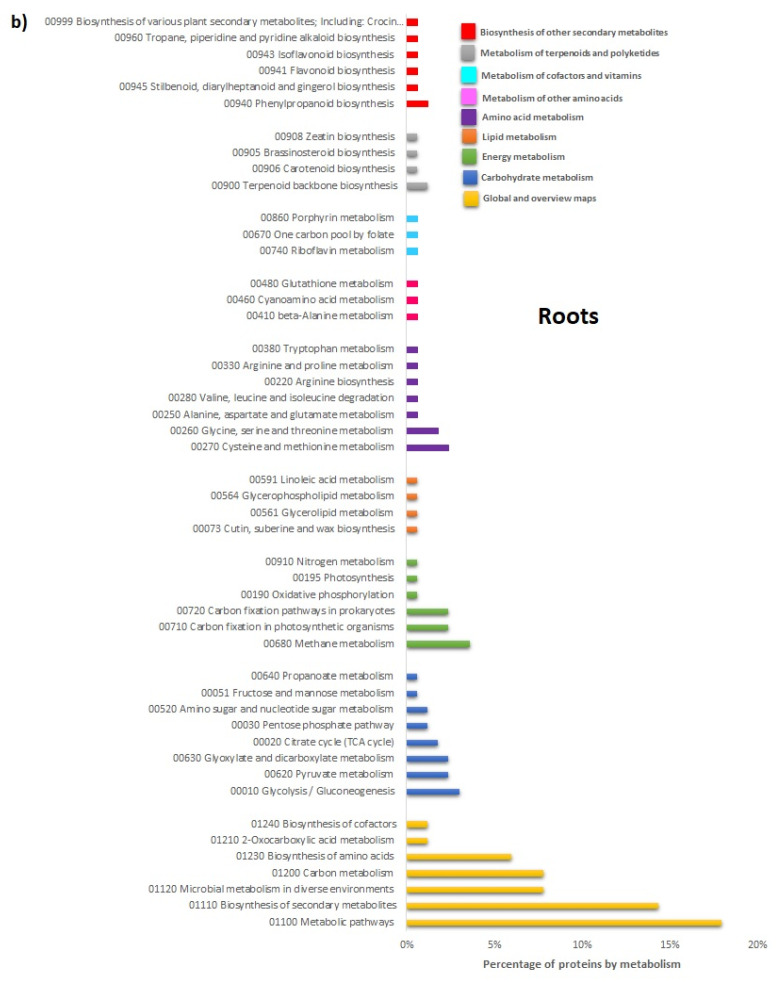
KEGG enrichment analysis of DAPs in the leaves (**a**) and roots (**b**) of *Lactuca sativa* seedlings after treatment with FCA.

To better understand the functions of DAPs, KEGG enrichment analyzes were also performed. The results showed that they could be assigned to 23 metabolic pathways in the leaves (Figure 6a) and 48 metabolic pathways in the roots, respectively. Furthermore, in the leaves, analysis revealed that the DAPs involved in specific metabolic pathways, especially the biosynthesis of secondary metabolites, oxidative phosphorylation, carbon metabolism, photosynthesis, carbon fixation, glyoxylate and dicarboxylate metabolism, and fructose and mannose metabolism were found to be the most enriched under the treatment (Figure 6a). Even in the roots, the biosynthesis of secondary metabolites, the microbial metabolism involving nitrogen metabolism, carbon metabolism, amino acid biosynthesis, methane metabolism, glycolysis, pyruvate biosynthesis, carbon fixation metabolism in photosynthetic prokaryotes, cysteine and methionine metabolism, and glycine, serine, and threonine metabolism were found to be the most enriched (Figure 6b). Further details are available in Supplementary Table S6.

#### 2.2.5. Overview of Protein Functional Analyses, GO, and KEGG Enrichment Analyzes

GO enrichment in the leaves categorized all DAPs mainly into the mitotic DNA damage response signaling, the S-adenosylmethionine biosynthetic process, glycolysis, photosynthesis, carbon fixation, photosynthetic electron transport in photosystem II, photorespiration, carbohydrate biosynthesis, and glycine metabolism (Figure 2a). As shown in GO classification, KEGG classification also found that carbon metabolism and carbohydrate metabolism were affected (Figure 6a). Most of the proteins that were of high abundance were found to belong to the Calvin cycle, photosynthesis, protein biosynthesis, and ATP metabolic process and translation; whereas proteins in the cell cycle, transcription, DNA repair, m-RNA binding, and signal transduction were in low abundance (Figure 5a). In roots, GO enrichment categorized all DAPs mainly into root cap development, lateral root formation and morphogenesis, glutamine biosynthesis, and S-adenosylmethionine metabolism (Figure 2b). KEGG classification found the metabolic pathway related to nitrogen metabolism, carbon metabolism, amino acid biosynthesis, methane metabolism, glycolysis, pyruvate biosynthesis, carbon fixation in photosynthetic prokaryotes, cysteine and methionine metabolism, and glycine, serine, and threonine metabolism. Proteins were in low abundance belonging to the response to water deprivation, one-carbon metabolic process, glutamine biosynthetic process, protein ubiquitination, and lateral root formation. Many other proteins were highly abundant in the cell cycle, defense response, root development, transmembrane transport, transcription, carbohydrates, and response to nitrogen starvation (Figure 6b).

## 3. Discussion

Application of organic fertilizers can significantly improve the growth, yield, and quality of the lettuce without negatively impacting the environment and human health [12]. Authors reported that, when composted urban waste was used, there were inhibitory or growth-inducing effects of the lettuce seedlings, possibly due to a combination of the high electrical conductivity, ammonia toxicity, and degree of stabilization of this compost [13,14]. Further investigations on the effects of the types of organic amendments on lettuce seedlings reported an enhanced plant biomass, and a higher content of the Rubisco large subunit and soluble proteins; on the contrary, an antagonistic effect was observed on the chlorophyll content [12].

Fast-composted soil amendment (FCA) produced by the urban, organic solid waste fractions and stabilized by the Fenton’s reaction procedure, revealed after 8 h of treatment, a good degree of oxidative decomposition with C/N ratios of 22, compared to an initial value of 26. In addition, the FCA exhibited a dark-brown color, was odorless, and had a humidity of 15%. The product cannot be considered as a conventional compost, but claimed to be a stabilized organic matrix. In this case, the material does not go, at least within 6 months, in further biochemical transformation when stored under dry conditions. Property of the material as a soil fertilizer was assessed on *Lactuca sativa* seedlings that underwent modification of the biochemical and molecular behavior; the FCA treatment, In fact, positively affected both root growth and photosynthesis. After 28 days treatment of *L. sativa* seedlings, the root biomass and chlorophyll content were found to have significantly increased with respect to the control seedlings. The chlorophyll physiological adjustments induced by the FCA treatment appeared to be linked to an increase in photosynthesis, as the proteins related to this metabolism accumulated in the shoots. Under our conditions, however, the shoot biomass was not affected by the treatment, indicating that the amendment exerted contrasting effects on shoot metabolism.

The FCA treatment also promoted primary and secondary metabolism in the leaves; the mitotic intra-S DNA damage checkpoint signaling process was enriched seventy times with respect to the untreated leaves; during the S-phase of the cell cycle, and in the event of problems during the replication process, DNA integrity checkpoints are activated slowing down the cell cycle to grant the cell time to repair the damage [15]; evidence has also suggested that this well-known mechanism has a role as a growth regulator processer in plants [16]. On this view, the FCA imposed a stress condition that, however, the leaf cells coped with by activating the mechanisms that slow down the mitosis to repair the DNA, this might lead to a slowdown in tissue growth.

Amendment also induced the enrichment of the S-adenosylmethionine (SAM) cycle; SAM is a key enzyme involved in many important biological processes, such as ethylene and polyamine biosynthesis, transmethylation, and transulfuration; SAM genes showed differential expression in response to abiotic stresses and exogenous hormone treatments [17]. Polyamines, for example, are important hormones that regulate cell growth during stress responses, pollen and flower development, and the protection of photosystem II [18]. The cell demand for SAM compounds, of course, may change markedly under different growth conditions with a metabolic cost of ATP consumption. In this regard, the metabolic processes of ATP production, mainly through photophosphorylation, have been enriched to cope with the high energy demand under treatment with FCA; the energy metabolism that uses carbohydrates was induced through the processes of photosynthetic gluconeogenesis, the biosynthesis of sucrose and glycolysis. To complete the effects on the metabolic patterns induced by the FCA treatment, the upregulation of processes involving the biosynthesis and metabolisms of glycine have been observed. It is well known that endogenous glycine accumulation mediates abiotic stress tolerance in plants involving the osmotic regulation [19]. In response to the FCA treatment, glycine should be synthesized in excess to adjust the osmotic stress induced by stress to maintain the sub-cellular structures and reduce the oxidative damage. In addition, the catabolic process of amino acids and dicarboxylic acids could be related to the high energy demand of treated leaves; catabolic pathways for several amino acids including alanine or glutamine are very short, and they can be directly converted to pyruvate by alanine aminotransferases and to glutamate, respectively, contributing substantially to the energy state of plant cells under certain physiological conditions [20].

In roots, the FCA treatment strongly enriched the organs morphogenesis and development; root cap development, lateral root formation, and post-embryonic root morphogenesis were the main biological processes enriched by the treatment. It is well known that morphogenetic processes are the basis of new organ formation; lateral roots morphogenesis is a decisive process during root system formation [21]. Regarding the root cap, it is the terminal tissue of the root of most plants. Historical evidence has shown that the root cap has not only the role of protecting proximal root meristem, but also to direct root growth in response to stimuli such as gravity, light, gradients in temperature, humidity, ions, and other chemicals [22]. Responses to water deprivation were strongly inhibited by the FCA treatment. The highly conserved plant aquaporins, known as plasma membrane intrinsic Proteins (PIPs), are the main gateways for cell membrane water exchange; in Arabidopsis, the inhibition of aquaporin expression in roots induced an increase in root growth [23]. Researchers have found that if plants lack aquaporins, thus having an increased resistance for the water movement in leaf and root cells, they compensate for this effect by increasing their root surface; this finding completes the evidence that FCA strongly alters the plant water transport and root growth dynamic. Additionally, the biosynthetic processes of amino acids, the biosynthesis of ribonucleotides, and the biosynthetic process of carboxylic acids have also been enriched. Morphological, biochemical, and molecular findings were, then, found to be completely consistent with the significant root growth promotion of lettuce seedlings cultivated on FCA-amended soil. FCA induced, in fact, the enrichment of the primary metabolism of purine, amino acids, and carbohydrates. Biological processes of the S-adenosylmethionine (SAM) cycle were strongly enriched. SAM, as reported above, is the main methyl group donor useful for the methylation of DNA, RNA, protein, lignin, and flavonoids, and it also plays important roles in regulating plant development under both abiotic or biotic stress [17], and heavy metal tolerance [24].

## 4. Materials and Methods

### 4.1. Fast-Composted Soil Amendment (FCA) Production

The fast-composted soil amendment (FCA) was produced starting from samples of municipal solid waste. Waste was dried under vacuum and finely ground; 200 gr of dried powder were placed in the glass reactor, Fe^2+^ (as the catalyst) at concentrations of 0.01% FeSO_4_ (VEBI Istituto Biochimico s.r.l., Borgoricco (PD), Italy) and 0.6 × 10^−3^ H_2_O_2_ (as the oxidant) (Panreac Applichem, Barcelona, Spain) were then added. The Fenton’s reaction was performed at pH 3.0, 60 °C, under a pressure of 0.96 bar for 8 h, during which the processes of chemical stabilization of the biomass took place. Further details of the reaction are reported in Roccotelli [25]. For the elemental analysis of FCA, the stabilized biomasses were subjected to high-temperature combustion (T ≥ 900 °C) in a bomb containing oxygen under pressure and following the method described in Nelms [26]; residues were analyzed by ICP/MS iCapQ (Thermofischer, Waltham, MA, USA) to evaluate the concentrations of C, H, and N in terms of their relative percentages. Results of the elemental analysis are reported in Supplementary Table S3.

### 4.2. Plant Acclimation, Treatments, and Growth

Seedlings of *Latuca sativa* L. var. longifolia (*n* = 70), with four leaves each, were transplanted in a soft and porous commercial soil, (Humifly, Humiflora, Italy) and mixed with pine bark, light peat, and volcanic lapilli in a proportion of 2:1 (*v*/*v*). Seedlings of *L. sativa* were acclimated under controlled temperatures and a short-day 10/14 h light/dark cycle. After seven days, each seedling was weighed and measured in length, and were then divided into three sub-cultivations of 35 seedlings each on soils whose compositions were as follows: (i) soil without amendment (control), and (ii) soil with 2% FCA [27]. After 30 days cultivation, all seedlings were collected; for each sub-cultivation, whole plant weight, weight of the roots and leaves, number of total leaves for each seedling, and the number of healthy leaves were measured. The leaves and roots were, then frozen in liquid nitrogen and stored at −80 °C to be used for the physiological and molecular analyzes.

### 4.3. Chlorophyll Extraction and Measurement

A total of 1.0 g of frozen leaf tissue was ground in liquid nitrogen in a mortar to obtain a fine powder; 5 mL of 80% cold acetone was added to the tissue powder and incubated at 4 °C for 3 h under weak shaking; then the samples were centrifuged at 800× *g* for 15 min. Then, 1 mL of crude supernatant was transferred in a cuvette and the absorbance was measured at 663 nm and 645 nm with the 7310 Jenway spectrophotometer. The concentrations of chlorophylls a and b, and of the total chlorophyll were determined by the following equations [28]:

(1) $$Chl\;a\;\left(\frac{\mathrm{mg}}{g}\right)=\frac{\left(12.7\;A_{663}-2.69\;\;A_{645}\right)\;\;0.01}{wt\;}$$

(2) $$Chl\;b\;\left(\frac{\mathrm{mg}}{g}\right)=\frac{\left(22.9\;\;A_{663}-4.68\;\;A_{645}\right)\;0.01}{wt}$$

(3) Chl tot=Chl a+Chl b

where chlorophylls content as mg/g fresh tissue were measured in six biological replicates.

### 4.4. Protein Extraction and Purification

#### 4.4.1. Protein Extraction and Purification from Leaves

Proteins from the leaves of three biological replicates of the control and FCA treatments were extracted by the multistep procedures [29]. For each extraction, 1.4 g of tissues were crushed in a mortar in liquid nitrogen until a fine powder was obtained. Ground plant tissue was homogenized with a volume of 10% TCA in acetone and centrifuged at 14,000× *g* for 5 min; a volume of 10% TCA in water was added and centrifuged at 14,000× *g* for 5 min. Subsequently, four washes were performed using 80% acetone in water. After centrifugation, the pellet containing the precipitated proteins was dried at room temperature. Approximately 100 mg of powdered tissue was dissolved in 0.8 mL of phenol (buffered with Tris HCL, pH8.0, Sigma, St. Louis, MO, USA) and 0.8 mL of SDS buffer (30% sucrose, 2% SDS, 0.1M Tris-HCl, pH8.0, 5% 2-mercaptoethanol) in a 2 mL microfuge tube. The samples were vortexed for 30 s and centrifuged at 14,000× *g* for 5 min to allow proteins to solubilize in the phenol phase. The phenol phase was mixed with five volumes of 0.1 M ammonium acetate in cold methanol, and then the mixture was stored at −20 °C for 30 min to precipitate the proteins. Proteins were collected by centrifugation at 14,000× *g* for 5 min. Two washes were then performed with 0.1 M ammonium acetate in cold methanol, and two with cold 80% acetone, and centrifuged at 14,000× *g* for 7 min. The final pellet containing purified protein was dried and dissolved in the Laemmli 1DE separation buffer overnight [30]. Proteins were then quantified by measuring the absorbance at 595 nm according to the Bradford assay [31]. Protein yield was calculated as the mg of protein for g fresh tissue weight of each biological replicate.

#### 4.4.2. Protein Extraction and Purification from Roots

Proteins from the roots of three biological replicates of the control and FCA treatments were extracted by 1 g of root tissue, weighed, and then pulverized in liquid nitrogen. A total of 1 mL of extraction buffer (0.7 M sucrose, 0.5 M Tris, 30 mM HCl, 50 mM EDTA, 0.1 M KCl, 2% 2-mercaptoethanol, 2 mM PMSF) was added to the pulverized tissue. The phenolic phase was performed by adding to the sample 500 µL of SDS Buffer (30% Sucrose, 2% SDS to be dissolved in 0.1 M Tris-HCl pH 8, 5% of 2-mercaptoethanol) and 500 µL of Phenol solution Sigma-Aldrich (equilibrated with 10 mM Tris HCl pH8 and 1 mM EDTA) [32]. The sample was shaken and centrifuged at 13,000 rpm for 8 min. The supernatant phenolic phase was recovered to which 0.1 M of ammonium acetate in cold methanol was added. The sample was placed at −20 °C for 30 min, then centrifuged at 13,000 rpm for 5 min. A second wash in ammonium acetate was performed, followed by two washes with 80% acetone. The final pellet was dried and then dissolved in the Laemmli 1DE separation buffer overnight [30]. Proteins were then quantified by measuring the absorbance at 595 nm according to the Bradford assay [31]. Protein yield was calculated as the mg of protein for g fresh tissue weight of each biological replicate.

### 4.5. SDS-PAGE Electrophoresis of Proteins in Gel Digestion, and Mass Spectrometry

A gel was prepared at a concentration of 12.5% in the running gel and 6% in the stacking gel of acrylamide/bisacrylamide, according to the method of Laemmli [30]. The samples were heated for 4 min at 100 °C before being loaded onto the gel. The electrophoretic run was conducted at 60 mA for the stacking gel and 120 mA in the running gel at a constant power of 200 V.

The electrophoresis ran for an average of 1 h and 30 min. The gels were stained with Coomassie Blue overnight and were subsequently destained with several changes of destaining solution (45% methanol, 10% acetic acid). Digitalized images of the SDS-PAGEs were analyzed by the Quantity One 1-D Analysis Software (Bio-Rad, Hercules, CA, USA) to measure the optical densities at each lane of all biological replicates. Each lane of the same SDS-PAGE was divided into six slices from 200 to10 kDa and were subsequently manually excised from the gel.

The CBB-stained gel slices were destained and then processed with the reduction (DTT) and alkylation (IAA) steps [33]. Gel pieces were digested by trypsin (Promega, Madison, WI, USA) overnight at 37 °C, followed by the addition of an ammonium bicarbonate buffer to cover the gel matrix. The extracted peptides were immediately processed for mass spectrometry analysis.

### 4.6. Mass Spectrometric Analysis

LC-MS/MS analysis was performed on an EASY-LC 1000 (Thermo Fisher Scientific, Odensem, Denmark) coupled to a hybrid quadrupole/Orbitrap Q-Exactive mass spectrometer (Thermo Fisher Scientific, Dreieich, Germany). An in-house made analytical column (length 14 cm, and inner diameter 75 μm) packed with 3 μm C 18 silica particles (Dr. Maisch, Entringen, Germany) was used. Samples were diluted 5-fold in 0.1% formic acid; then, 2 μL of the resulting peptide mix was injected for LC-MS/MS analysis. Mobile phase A was 2% acetonitrile, 0.1% formic acid; mobile phase B was 80% acetonitrile, 0.1% formic acid. The LC mobile phase composition went from 0% mobile phase B to 3% mobile phase B in 1 s, then from 3 to 40% B in 120 min, and then to 100% B following an additional 8 min; after 5 min at 100% B, the mobile phase composition was brought back to 0% B in 2 min, for a total run time of 135 min at a flow rate of 230 nL/min. The column effluent was subjected to nano-electrospray ionization (1600 V of nESI potential), and the resulting charged species were detected by the Q-Exactive hybrid mass spectrometer operating in the positive ion mode. A full MS scan was acquired in the Orbitrap analyzer at a resolution of 70,000, *m*/*z* range of 350–1800, and target AGC value of 1.00 × 10^−6^, respectively, The data-dependent MS/MS acquisition (DDA) procedure was performed by selecting the 12 most abundant peaks with more than two charges after each full scan analysis (top 12 method). Precursor ions were fragmented by HCD (high-energy collisional dissociation); HCD normalized collision energy was calculated as 25%. MS/MS analysis was conducted in the Orbitrap analyzer at a resolution of 35,000,target AGC value of 1.0 × 10^−5^, and an intensity threshold of 5.0 × 10^−4^; the isolation window was set to 1.6 *m*/*z*. A maximum injection time of 50 ms was set for the full MS scan event, while 120 ms was the maximum injection time allowed for tandem MS/MS scans. Dynamic exclusion time was set to 30 s.

### 4.7. Bioinformatic Analysis and Proteins Identification

From the MS/MS spectra, protein inference and validation were performed with the Scaffold software 4.8 (Proteome Software, Inc., Portland, OR, USA). MS/MS spectra were extracted from the raw data by accepting one minimum sequence of eight amino acids, and fusion scans with the same precursor within one mass window of ±0.4 *m*/*z* over a time interval of ±30 s. The key parameters of research were the scored peak intensity, (SPI) ≥ 50%, the precursor mass tolerance of ±10 ppm, and the mass tolerance of product ions of ±20 ppm. The carbamidomethylating of cysteine was fixed as a modification, and trypsin was selected as the enzyme for the digestion, accepting two missing cleavages per peptide.

The automatic thresholds were used for peptide identification in the software Scaffold. Generally, peptide probabilities are evaluated using a Bayesian approach for the estimation of the local FDR (LFDR) up to a value of 1%. The peptide sequences using the Scaffold 4.8 Q+S system software were interfaced with both the database of proteins deduced from the generalist protein sequences of *Lactuca sativa* deposited in the NCBI database (downloaded in June 2022) and in the bank UniProt data (downloaded in June 2022). Identified peptides assigned to each protein in all samples and the related statistical parameters for significant identification are reported in Supplementary Tables S2 and S3.

### 4.8. Semi-Quantitative Analysis of Identified Proteins

Three biological replicates of leaves and three of roots for each test were used for quantitative analyzes. The relative abundance of proteins among the samples was performed by choosing the label free quantitative method “Total Spectra” from the Quantitative menu of the Scaffold software (Proteome Software, Inc., Portland, OR, USA; version 5.1). This method uses the sum of all weighted spectra that are associated with a specific protein and within a sample, where the weight is a measure of how much a spectrum is shared by other proteins. Spectral count was undertaken only on statistically validated spectra to increase its accuracy. Consequently, it was used for quantitation comparisons. A peptide with less than two matches was discarded. The missing values were considered undetectable and were thus assumed that they were under the limit of detection, but still present. Thus, when they were undetectable, a zero value was attributed, and they were considered in the statistical calculation. To identify the proteins which show different quantitative abundances in two or more categories, the test “Fold change by category” was used. Fold change (FC) is expressed as the ratio of the quantitative value in one sample (or category) over the quantitative value in a second sample. Values of FC > 1 indicate a high quantitative profile and protein results as accumulated, while values of FC < 1 indicate a low quantitative profile and protein results as depleted. As the specified minimum value replaces any missing values, if a zero appears in the denominator an INF will appear in the FC column. The FC has also been log2 normalized for this study, and analysis was conducted using a threshold of 2 for the significance of the quantitative profile.

### 4.9. Gene Ontology Categories and PANTHER and KEGG Pathway Enrichment Analysis

Gene ontology (GO) categories of all differential accumulated proteins were assigned by means of the panther classification system [34]; then, a statistical assessment of the differences in functional classes between two groups of sequences based on the Fisher test analysis were executed against the complete dataset of sequences of *Lactuca sativa*. Three different significance parameters are given for the false-positive control: false discovery rate (FDR), family-wise error rate (FWER), and a single test *p*-value (Fisher *p*-value). By taking an FDR significance threshold of 0.05, we obtain functionalities that are specific for the organs and significant for the proteins in the treated leaves and roots. The relative fold-change of each GO term has been represented for the “Biological process” categories.

The list of FASTA sequences of DAPs whose variation patterns were highly significant (*p* < 0.05) were subjected to BlastKOALA (http://www.kegg.jp/blastkoala) (accessed on 27 May 2023) [35] analysis to obtain KEGG Orthology (KO) assignments. The KO identifier (called the K number) list was then used for KEGG pathway mapping through the KEGG mapper web server (http://www.genome.jp/kegg/tool/map_pathway2.html) (accessed on 27 May 2023) [36].

### 4.10. Statistics

Comparison of the differences among the groups of values for the biomass and photosynthetic pigment were analyzed using the t-test with a *p* < 0.05 threshold for statistical significance. All the statistical analyzes were performed using Excel XLSTAT (©Addinsoft, Paris, France, released at 2022.6.1.1187). Significance was defined as *p* ≤ 0.05.

For the proteomics results, comparison of differences among the groups was conducted using the differentially expression and heat map tools available at XLSTAT. The Bonferroni test was used to test for the assumption of homogeneity of variances. Threshold for significance was *p* ≤ 0.05.

## 5. Conclusions

**Figure 7:**
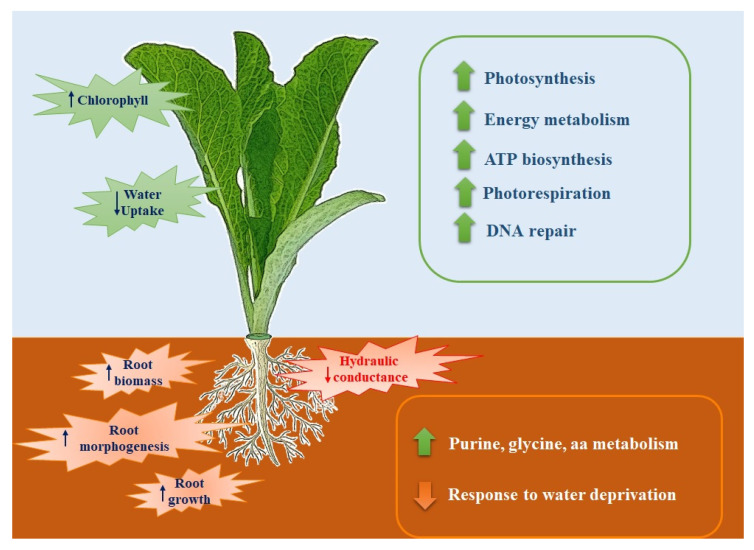
Illustration showing the responses of *Lactuca sativa* seedlings to FCA treatment. Increased activity or accumulation of the relevant process or metabolism is depicted by upward arrows, whereas decreased activity or depletion of the relevant process is depicted by downward arrows.

Fast-composted soil amendment treatment played an important role in the growth and differentiation of lettuce roots. The addition of the fast-composted soil amendment to the soil seemed to have a lower impact on leaf development that adjusted the metabolism by increasing the energy demand to cope with the effects imposed by the treatment. Seedlings grown on amended soil had, in fact, a higher root biomass, and a higher chlorophyll content, but not a higher leaf biomass compared to the seedlings grown on regular soil. Proteomic analyzes confirmed these positive effects as the increase in primary production and primary metabolism occurred in the roots with a variation in proteins involved in energy metabolism, root formation, osmotic regulation, and water balance in the root-shoot system (Figure 7). Even if the treatment was conducted for a short period, taken altogether, our results demonstrated that the fast-composted soil amendment produced by the method based on the Fenton’s reaction had positive effects on *Lactuca sativa,* and strongly suggest that it could be used in intensive crop cultivation, thus contributing to the sustainability of agronomic practices, and accelerating the recycling of municipal solid waste.

## Data Availability

The data presented in this study are available at https://www.resifac.com, accessed on 3 June 2023.

## Supplementary Materials

The following supporting information can be downloaded at: https://www.mdpi.com/article/10.3390/plants12122234/s1, Table S1: (a) Differentially accumulated proteins (DAPs) identified in *L. sativa* leaves treated with FCA amendment. Accession number, protein name, fold change reported also as Log2 value, biological process whose DAPs are belonging were reported, (b) Differentially accumulated proteins (DAPs) identified in *L. sativa* leaves treated with FCA amendment. Protein name, accession number, number of the exclusive MS/MS spectra assigned to each protein in each biological replicate, (c) Differentially accumulated proteins (DAPs) identified in *L. sativa* leaves treated with FCA amendment and significance in their variation among samples and replicates, (d) Heat map values dataset and SD, (e) Sequence enrichment in *L. sativa* samples treated with FCA; Table S2: (a) Differentially accumulated proteins (DAPs) identified in *L. sativa* roots treated with FCA amendment. Accession number, protein name, fold change reported also as Log2 value, biological process whose DAPs are belonging were reported, (b) Differentially accumulated proteins (DAPs) identified in *L. sativa* roots treated with FCA amendment. Protein name, Accession number, number of exclusive MS/MS spectra assigned to each protein in each biological replicate, (c) Differentially accumulated proteins (DAPs) identified in *L. sativa* roots treated with FCA amendment and significance of their pattern of variation among samples and replicates, (d) Heat map values dataset, (e) Sequence enrichment assigned to GO biological process in *L. sativa* roots treated with FCA; Table S3: Elemental HCN analysis. Percentage of each element in: (A) original dried organic waste; (B) dried Fenton reacted organic waste; Table S4: Identified peptides assigned to each protein in all samples of leaves and the related statistical parameters for significant identification; Table S5: Identified peptides assigned to each protein in all samples of roots and the related statistical parameters for significant identification; Table S6: (a) KEGG enrichment analysis of DAPs in roots of *Lactuca sativa* seedlings after treatment with FCA, (b) KEGG enrichment analysis of DAPs in leaves of *Lactuca sativa* seedlings after treatment with FCA.
