# Supplementary material for: Growth and Primary Metabolism of Lettuce Seedlings (Lactuca sativa L.) Are Promoted by an Innovative Iron-Based Fenton-Composted Amendment
Source: Plants (Basel). 2023 Jun 7;12(12):2234. doi: 10.3390/plants12122234 (PMC10301099; doi:10.3390/plants12122234)
Supplement: Supplementary file 1 [file plants-12-02234-s001.zip › Table S6.pdf]

"**Research paper:** Growth and primary metabolism of lettuce seedlings (*Lactuca sativa* L.) are promoted by an innovative Iron-Based Fenton composted amendment.
Amalia Piro, Daniela Oliva, Dante Matteo Nisticò, Ilaria Lania, Maria Rita Basile, Giuseppe Chidichimo, Silvia Mazzuca"

Supplementary table S6a. KEGG enrichment analysis of DAPs in roots of *Lactuca sativa* seedlings after treatment with FCA

Query	KO	Definition
sp|O64989|C90B1_ARATH (513)	K09587	CYP90B, DWF4; steroid 22S-hydroxylase [EC:1.14.14.178]
sp|Q3MV14|DSE1_ARATH (386)	K24770	DSE1, ALT2, EMB2757; protein decreased size exclusion limit 1
sp|Q6F3F1|METK4_ATRNU (396)	K00789	metK, MAT; S-adenosylmethionine synthetase [EC:2.5.1.6]
sp|Q9SCY2|FKB13_ARATH (208)	K01802	E5.2.1.8; peptidylprolyl isomerase [EC:5.2.1.8]
sp|Q8RXG3|M2K5_ARATH (348)	K13413	MKK4_5; mitogen-activated protein kinase kinase 4/5 [EC:2.7.12.2]
sp|Q9XIF2|MTR4_ARATH (988)	K12598	MTR4, SKIV2L2; ATP-dependent RNA helicase DOB1 [EC:3.6.4.13]
sp|Q9FX68|ZWIP6_ARATH (302)		
sp|Q8RWH9|NUP58_ARATH (513)	K14307	NUPL1, NUP49; nucleoporin p58/p45
sp|Q9SLK2|ALIS3_ARATH (349)		
sp|Q5K4R0|MAD47_ORYSJ (250)		
sp|Q9FME5|COBL5_ARATH (204)		
sp|Q96552|METK2_CATRO (393)	K00789	metK, MAT; S-adenosylmethionine synthetase [EC:2.5.1.6]
sp|Q9LNQ1|TI231_ARATH (187)	K17794	TIM23; mitochondrial import inner membrane translocase subunit TIM23
sp|Q8GYT9|SIS3_ARATH (358)	K16284	SIS3; E3 ubiquitin-protein ligase SIS3 [EC:2.3.2.27]
sp|Q9CA93|BAC2_ARATH (296)	K15109	SLC25A20_29, CACT, CACL, CRC1; solute carrier family 25 (mitochondrial carnitine/acylcarnitine transporter), member 20/29
sp|Q38841|AGL12_ARATH (211)		
sp|Q84WW2|6PGL3_ARATH (325)	K01057	PGLS, pgl, devB; 6-phosphogluconolactonase [EC:3.1.1.31]
sp|Q6NKU9|TI223_ARATH (214)	K17790	TIM22; mitochondrial import inner membrane translocase subunit TIM22
sp|Q9SJQ1|PXC1_ARATH (672)		
sp|Q5H8A6|CASTO_LOTJA (853)	K21866	POLLUX, DMI1, CASTOR; ion channel POLLUX/CASTOR
sp|O23254|GLYC4_ARATH (471)	K00600	glyA, SHMT; glycine hydroxymethyltransferase [EC:2.1.2.1]

sp|Q96551|METK1_CATRO (393)	K00789	metK, MAT; S-adenosylmethionine synthetase [EC:2.5.1.6]
sp|P13868|CALM1_SOLTU (149)	K02183	CALM; calmodulin
sp|Q9STE8|TC753_ARATH (818)		
sp|Q9LIK0|PKP1_ARATH (596)	K00873	PK, pyk; pyruvate kinase [EC:2.7.1.40]
sp|Q8L7W2|NUDT8_ARATH (369)		
sp|Q94A28|ACO2M_ARATH (995)	K01681	ACO, acnA; aconitate hydratase [EC:4.2.1.3]
sp|Q67Z52|TBCB_ARATH (243)	K17262	TBCB, CKAP1, ALF1; tubulin-specific chaperone B
sp|O24520|HBL1_ARATH (160)		
sp|Q9ZT81|CSPLK_ARATH (164)		
sp|Q37001|1A15_ARATH (470)	K01762	ACS; 1-aminocyclopropane-1-carboxylate synthase [EC:4.4.1.14]
sp|Q9LE81|IRE_ARATH (1168)		
sp|Q9AVJ9|MXMT1_COFAR (378)	K21482	BAMT; benzoate O-methyltransferase [EC:2.1.1.273]
sp|Q5W7C1|STAR2_ORYSJ (285)	K02069	STAR2, fetB; UDP-glucose/iron transport system permease protein
sp|Q9S7Z3|PCS1_ARATH (485)	K05941	E2.3.2.15; glutathione gamma-glutamylcysteinyltransferase [EC:2.3.2.15]
	K25587	E3.4.17.25; glutathione-S-conjugate glycine hydrolase [EC:3.4.17.25]
sp|Q9LSF6|VTH21_ARATH (219)	K22736	VIT; vacuolar iron transporter family protein
sp|Q766Z3|REV3_ARATH (1890)	K02350	REV3L, POLZ; DNA polymerase zeta [EC:2.7.7.7]
sp|Q8S8S1|PEX16_ARATH (367)	K13335	PEX16; peroxin-16
sp|O24379|LOX12_SOLTU (861)	K15718	LOX1_5; linoleate 9S-lipoxygenase [EC:1.13.11.58]
sp|B9G5Y5|AB25G_ORYSJ (1004)		
sp|O49485|SERA1_ARATH (603)	K00058	serA, PHGDH; D-3-phosphoglycerate dehydrogenase / 2-oxoglutarate reductase [EC:1.1.1.95 1.1.1.399]
sp|Q8RXE1|GAUT5_ARATH (610)	K13648	GAUT; alpha-1,4-galacturonosyltransferase [EC:2.4.1.43]
sp|O23180|PLP5_ARATH (414)		
sp|Q0WQG8|GTG2_ARATH (467)		
sp|P43281|METK2_SOLLC (393)	K00789	metK, MAT; S-adenosylmethionine synthetase [EC:2.5.1.6]
sp|Q8LDP4|CP19D_ARATH (201)	K01802	E5.2.1.8; peptidylprolyl isomerase [EC:5.2.1.8]

sp|P22221|PPDK_FLATR (953)	K01006	ppdK; pyruvate, orthophosphate dikinase [EC:2.7.9.1]
sp|O04089|ZIP4_ARATH (374)	K14709	SLC39A1_2_3, ZIP1_2_3; solute carrier family 39 (zinc transporter), member 1/2/3
sp|Q9LZW4|CIPKE_ARATH (442)		
sp|Q7XZR1|METK1_ATRNU (396)	K00789	metK, MAT; S-adenosylmethionine synthetase [EC:2.5.1.6]
sp|Q652K1|SGR_ORYSJ (274)	K22013	SGR, SGRL; magnesium dechelatase [EC:4.99.1.10]
sp|Q9LDM2|CER7L_ARATH (307)	K03678	RRP45, EXOSC9; exosome complex component RRP45
sp|Q10MI0|SRL2_ORYSJ (988)	K21842	EFR3; protein EFR3
sp|Q9LMA7|CXE1_ARATH (318)		
sp|Q9AST8|ROSY1_ARATH (160)		
sp|Q9CAU2|SCP5_ARATH (438)	K16296	SCPL-I; serine carboxypeptidase-like clade I [EC:3.4.16.-]
sp|Q5GHF7|IPT_HUMLU (329)	K10760	IPT; adenylate dimethylallyltransferase (cytokinin synthase) [EC:2.5.1.27 2.5.1.112]
sp|Q9SEZ1|ZHD11_ARATH (242)		
sp|Q9LW00|MBD11_ARATH (254)		
sp|H6WS93|PDR1_PETAX (1452)	K08711	PDR, CDR1; ATP-binding cassette, subfamily G (WHITE), member 2, PDR
sp|Q9LFS4|NIK1_ARATH (638)		
sp|Q6XMI3|BSMT1_ARATH (379)	K21483	SAMT; salicylate 1-O-methyltransferase [EC:2.1.1.274]
sp|Q0WQF4|VP53A_ARATH (828)	K20299	VPS53; vacuolar protein sorting-associated protein 53
sp|Q9SN68|RAF2B_ARATH (200)	K07889	RAB5C; Ras-related protein Rab-5C
sp|Q949P1|ABAH1_ARATH (467)	K09843	CYP707A; (+)-abscisic acid 8'-hydroxylase [EC:1.14.14.137]
sp|O65530|PEK14_ARATH (731)		
sp|Q06611|PIP12_ARATH (286)	K09872	PIP; aquaporin PIP
sp|Q69XJ0|SPX1_ORYSJ (295)		
sp|Q9ZWS9|ARR3_ARATH (231)	K14492	ARR-A; two-component response regulator ARR-A family
sp|Q9FUY7|C79F2_ARATH (537)		
sp|Q94C74|GLYM2_ARATH (517)	K00600	glyA, SHMT; glycine hydroxymethyltransferase [EC:2.1.2.1]
sp|O82345|BAG6_ARATH (1043)		

sp|P17784|ALFC1_ORYSJ (358)	K01623	ALDO; fructose-bisphosphate aldolase, class I [EC:4.1.2.13]
sp|Q06197|IDHC_SOYBN (413)	K00031	IDH1, IDH2, icd; isocitrate dehydrogenase [EC:1.1.1.42]
sp|P53392|SUT2_STYHA (662)	K17470	SULTR1; sulfate transporter 1, high-affinity
sp|Q7XHW5|C14B1_ORYSJ (534)	K20661	CYP714A1; cytochrome P450 family 714 subfamily A1
sp|Q0WT24|STOP2_ARATH (373)		
sp|Q6ZIB5|PIN5C_ORYSJ (370)	K13947	PIN; auxin efflux carrier family protein
sp|Q8S341|PPA7_ARATH (328)	K14379	ACP5; tartrate-resistant acid phosphatase type 5 [EC:3.1.3.2]
sp|Q1H595|LSM2_ARATH (93)	K12621	LSM2; U6 snRNA-associated Sm-like protein LSm2
sp|Q84S07|NIP33_ORYSJ (278)	K09874	NIP; aquaporin NIP
sp|Q94A57|PHL2_ARATH (295)		
sp|Q852M4|PLAT3_ORYSJ (749)	K01301	NAALAD; N-acetylated-alpha-linked acidic dipeptidase [EC:3.4.17.21]
sp|Q8S397|NHX4_ARATH (529)		
sp|Q9SXF8|PIP13_ORYSJ (288)	K09872	PIP; aquaporin PIP
sp|A8MRY9|UGNT1_ARATH (344)	K15281	SLC35D1_2_3; solute carrier family 35, member D1/2/3
sp|Q37145|ACA1_ARATH (1020)	K01537	ATP2C; P-type Ca2+ transporter type 2C [EC:7.2.2.10]
sp|A0A1D6HQ92|YCED1_MAIZE (293)		
sp|Q0D9R7|ARFS_ORYSJ (1161)		
sp|Q9SFU3|PPA15_ARATH (532)	K22390	ACP7; acid phosphatase type 7
sp|Q9XFR0|KCO3_ARATH (260)	K05389	KCNKF; potassium channel subfamily K, other eukaryote
sp|P04770|GLNA1_PHAVU (356)	K01915	glnA, GLUL; glutamine synthetase [EC:6.3.1.2]
sp|Q93Z81|CAX3_ARATH (459)	K07300	chaA, CAX; Ca2+:H+ antiporter
sp|Q50LH3|C7192_ESCCA (495)	K09754	CYP98A, C3'H; 5-O-(4-coumaroyl)-D-quinate 3'-monooxygenase [EC:1.14.14.96]
sp|Q94A16|CP21C_ARATH (230)		
sp|Q94FA5|METK4_BRAJU (390)	K00789	metK, MAT; S-adenosylmethionine synthetase [EC:2.5.1.6]
sp|Q945Q1|CYT1_ARATH (101)		
sp|Q8H0V4|DUF7_ARATH (722)		

sp|Q8GVE8|CAPP4_ARATH (1032)	K01595	ppc; phosphoenolpyruvate carboxylase [EC:4.1.1.31]
sp|Q9FMF5|RPT3_ARATH (746)		
sp|P43282|METK3_SOLLC (390)	K00789	metK, MAT; S-adenosylmethionine synthetase [EC:2.5.1.6]
sp|Q40872|AG_PANGI (242)	K09264	K09264; MADS-box transcription factor, plant
sp|Q9FMS7|OFP10_ARATH (196)		
sp|Q08062|MDHC_MAIZE (332)	K00025	MDH1; malate dehydrogenase [EC:1.1.1.37]
sp|O81001|GRF1_ARATH (530)		
sp|Q8GXJ4|GLR34_ARATH (959)	K05387	GRIP; glutamate receptor, ionotropic, plant
sp|Q9FFN7|DGK2_ARATH (712)	K00901	dgkA, DGK; diacylglycerol kinase (ATP) [EC:2.7.1.107]
sp|Q7XSQ9|PIP12_ORYSJ (288)	K09872	PIP; aquaporin PIP
tr|L0GDQ5|L0GDQ5_WHEAT (262)	K06630	YWHAE; 14-3-3 protein epsilon
tr|A0A0U2WTZ1|A0A0U2WTZ1_PRUAV (290)	K09872	PIP; aquaporin PIP
sp|Q96293|ACT8_ARATH (377)	K10355	ACTF; actin, other eukaryote
tr|A0A178WDV5|A0A178WDV5_ARATH (377)	K10355	ACTF; actin, other eukaryote
tr|A0A140JW78|A0A140JW78_9GENT (304)	K09422	MYBP; transcription factor MYB, plant
sp|Q9LHJ9|P2C38_ARATH (385)	K01102	PDP; pyruvate dehydrogenase phosphatase [EC:3.1.3.43]
tr|B9DH70|B9DH70_ARATH (337)		
tr|B6TD48|B6TD48_MAIZE (307)	K00472	P4HA; prolyl 4-hydroxylase [EC:1.14.11.2]
tr|A5YM78|A5YM78_CICAR (261)	K06630	YWHAE; 14-3-3 protein epsilon
tr|Q0WTE4|Q0WTE4_ARATH (186)	K08515	VAMP7; vesicle-associated membrane protein 7
tr|Q705X3|Q705X3_MEDTR (233)	K12462	ARHGDI, RHOGDI; Rho GDP-dissociation inhibitor
tr|Q7Y0W8|Q7Y0W8_LUPAL (412)	K00031	IDH1, IDH2, icd; isocitrate dehydrogenase [EC:1.1.1.42]
tr|Q9XGB4|Q9XGB4_TRIRP (298)	K01183	E3.2.1.14; chitinase [EC:3.2.1.14]
tr|A0A2K8GLT3|A0A2K8GLT3_LUPAL (487)	K03327	TC.MATE, SLC47A, norM, mdtK, dinF; multidrug resistance protein, MATE family
tr|F8WL60|F8WL60_RHISY (297)	K10355	ACTF; actin, other eukaryote
tr|A0A1U8YLW4|A0A1U8YLW4_VIGUN (289)	K09872	PIP; aquaporin PIP

tr|A0A076V5N5|A0A076V5N5_9APIA (509)	K00938	E2.7.4.2, mvaK2; phosphomevalonate kinase [EC:2.7.4.2]
tr|B2L2W9|B2L2W9_9SOLA (260)	K08081	TR1; tropinone reductase I [EC:1.1.1.206]
tr|A0A1U8YLV7|A0A1U8YLV7_VIGUN (289)	K09872	PIP; aquaporin PIP
tr|Q9SAZ6|Q9SAZ6_MAIZE (960)	K01595	ppc; phosphoenolpyruvate carboxylase [EC:4.1.1.31]
tr|I3NN78|I3NN78_SOLLC (310)		
tr|V5RDW6|V5RDW6_CUCSA (292)	K09872	PIP; aquaporin PIP
tr|L7NJI5|L7NJI5_GOSBA (267)	K01183	E3.2.1.14; chitinase [EC:3.2.1.14]
tr|C0Z2S6|C0Z2S6_ARATH (325)	K13260	CYP81E; isoflavone/4'-methoxyisoflavone 2'-hydroxylase [EC:1.14.14.90 1.14.14.89]
tr|B2ZUU2|B2ZUU2_LOTJA (516)		
tr|A0A2Z5EMU8|A0A2Z5EMU8_ORYSI (568)		
tr|A0A0D5W5Q1|A0A0D5W5Q1_9ROSI (287)	K09872	PIP; aquaporin PIP
tr|M4M6J9|M4M6J9_TRIRP (182)		
sp|Q42908|PMGI_MESCR (559)	K15633	gpmI; 2,3-bisphosphoglycerate-independent phosphoglycerate mutase [EC:5.4.2.12]
sp|P14654|GLN12_ORYSJ (357)	K01915	glnA, GLUL; glutamine synthetase [EC:6.3.1.2]
tr|C5G5Y5|C5G5Y5_PHAAN (301)	K08235	E2.4.1.207; xyloglucan:xyloglucosyl transferase [EC:2.4.1.207]
tr|O22339|O22339_MEDSA (290)	K09872	PIP; aquaporin PIP
tr|Q3T1V7|Q3T1V7_9LILI (517)	K20000	MATK; maturase K
sp|Q42899|GLNA1_LOTJA (356)	K01915	glnA, GLUL; glutamine synthetase [EC:6.3.1.2]
tr|B6SM21|B6SM21_MAIZE (330)	K08064	NFYA, HAP2; nuclear transcription factor Y, alpha
sp|Q10M12|40C1_ORYSJ (348)		
tr|M1EY53|M1EY53_9ROSI (281)	K09872	PIP; aquaporin PIP
sp|P42895|ENO2_MAIZE (446)	K01689	ENO, eno; enolase [EC:4.2.1.11]
tr|Q41730|Q41730_MAIZE (169)		
tr|Q1H8M9|Q1H8M9_BETVU (290)		
tr|H2BPE1|H2BPE1_9MONI (427)	K02112	ATPF1B, atpD; F-type H+/Na+-transporting ATPase subunit beta [EC:7.1.2.2 7.2.2.1]
tr|A0A804J464|A0A804J464_MUSAM (386)	K05605	HIBCH; 3-hydroxyisobutyryl-CoA hydrolase [EC:3.1.2.4]

tr|I1SUZ0|I1SUZ0_CICAR (261)	K06630	YWHAE; 14-3-3 protein epsilon
tr|T1R3E6|T1R3E6_DATGL (187)		
tr|A0A075EC73|A0A075EC73_ASTME (571)	K00021	HMGCR; hydroxymethylglutaryl-CoA reductase (NADPH) [EC:1.1.1.34]
tr|A0A2H4RK81|A0A2H4RK81_9ERIC (1223)		
tr|Q5KTN5|Q5KTN5_TOBAC (260)	K06630	YWHAE; 14-3-3 protein epsilon
tr|L0GED8|L0GED8_WHEAT (261)	K06630	YWHAE; 14-3-3 protein epsilon
tr|L7X5Z3|L7X5Z3_GOSHI (246)		
XP_009387221.1 (277)	K08150	SLC2A13, ITR; MFS transporter, SP family, solute carrier family 2 (myo-inositol transporter), member 13
XP_018677474.1 (264)	K13066	COMT; caffeic acid 3-O-methyltransferase / acetylserotonin O-methyltransferase [EC:2.1.1.68 2.1.1.4]
XP_018673750.1 (335)	K13356	FAR; alcohol-forming fatty acyl-CoA reductase [EC:1.2.1.84]

"Research paper: Growth and primary metabolism of lettuce seedlings (*Lactuca sativa* L.) are promoted by an innovative Iron-Based Fenton composted amendment.
Amalia Piro, Daniela Oliva, Dante Matteo Nisticò, Ilaria Lania, Maria Rita Basile, Giuseppe Chidichimo, Silvia Mazzuca**"**

Supplementary table S6b. KEGG enrichment analysis of DAPs in leaves of *Lactuca sativa* seedlings after treatment with FCA

Query	KO	Definition
tr|A0A2J6KJN7|A0A2J6KJN7_LACSA (496)	K02112	ATPF1B, atpD; F-type H+/Na+-transporting ATPase subunit beta [EC:7.1.2.2 7.2.2.1]
tr|A0A2J6KR38|A0A2J6KR38_LACSA (484)	K01601	rbcL, cbbL; ribulose-bisphosphate carboxylase large chain [EC:4.1.1.39]
tr|A0A2J6K870|A0A2J6K870_LACSA (648)	K02132	ATPeF1A, ATP5A1, ATP1; F-type H+-transporting ATPase subunit alpha
tr|A0A2J6KSN5|A0A2J6KSN5_LACSA (609)		
tr|A0A2J6KR44|A0A2J6KR44_LACSA (329)	K02716	psbO; photosystem II oxygen-evolving enhancer protein 1
tr|A0A2J6KNE7|A0A2J6KNE7_LACSA (949)	K08675	PRSS15, PIM1; ATP-dependent Lon protease [EC:3.4.21.53]
tr|A0A2J6KM75|A0A2J6KM75_LACSA (377)	K00847	E2.7.1.4, scrK; fructokinase [EC:2.7.1.4]
tr|A0A2J6KKS5|A0A2J6KKS5_LACSA (955)	K01535	PMA1, PMA2; H+-transporting ATPase [EC:7.1.2.1]
tr|A0A2J6KC04|A0A2J6KC04_LACSA (328)	K00025	MDH1; malate dehydrogenase [EC:1.1.1.37]
tr|A0A2J6JYJ9|A0A2J6JYJ9_LACSA (221)	K02995	RP-S8e, RPS8; small subunit ribosomal protein S8e

tr|A0A2J6M0L9|A0A2J6M0L9_LACSA (647)	K03283	HSPA1s; heat shock 70kDa protein 1/2/6/8
tr|A0A2J6LQR8|A0A2J6LQR8_LACSA (450)	K07374	TUBA; tubulin alpha
tr|A0A2J6K4N9|A0A2J6K4N9_LACSA (233)	K02934	RP-L6e, RPL6; large subunit ribosomal protein L6e
tr|A0A2J6K1M5|A0A2J6K1M5_LACSA (1252)		
tr|A0A2J6K1D0|A0A2J6K1D0_LACSA (813)		
tr|A0A2J6JZ15|A0A2J6JZ15_LACSA (382)		
tr|A0A2J6JZ00|A0A2J6JZ00_LACSA (471)		
tr|A0A2J6K6R8|A0A2J6K6R8_LACSA (431)	K11340	ACTL6A, INO80K; actin-like protein 6A
tr|A0A2J6JYV6|A0A2J6JYV6_LACSA (843)	K03234	EEF2; elongation factor 2
tr|A0A2J6LKC2|A0A2J6LKC2_LACSA (190)	K11254	H4; histone H4
tr|A0A2J6JPA2|A0A2J6JPA2_LACSA (203)	K12876	RBM8A, Y14; RNA-binding protein 8A
tr|A0A2J6MF89|A0A2J6MF89_LACSA (99)	K02692	psaD; photosystem I subunit II
tr|A0A2J6JN28|A0A2J6JN28_LACSA (777)	K19984	EXOC5, SEC10; exocyst complex component 5
tr|A0A2J6K212|A0A2J6K212_LACSA (266)	K08912	LHCB1; light-harvesting complex II chlorophyll a/b binding protein 1
tr|A0A2J6JGN9|A0A2J6JGN9_LACSA (1409)	K15601	KDM3; [histone H3]-dimethyl-L-lysine9 demethylase [EC:1.14.11.65]
tr|A0A2J6M6Y2|A0A2J6M6Y2_LACSA (218)	K03564	BCP, PRXQ, DOT5; thioredoxin-dependent peroxiredoxin [EC:1.11.1.24]
tr|A0A2J6M0G5|A0A2J6M0G5_LACSA (523)		
tr|A0A2J6MHG7|A0A2J6MHG7_LACSA (464)	K01369	LGMN; legumain [EC:3.4.22.34]
tr|A0A2J6LS26|A0A2J6LS26_LACSA (1150)	K03021	RPC2, POLR3B; DNA-directed RNA polymerase III subunit RPC2 [EC:2.7.7.6]
tr|A0A2J6KRH0|A0A2J6KRH0_LACSA (403)	K02930	RP-L4e, RPL4; large subunit ribosomal protein L4e
tr|A0A2J6MHA8|A0A2J6MHA8_LACSA (957)	K10405	KIFC1; kinesin family member C1
tr|A0A2J6LNZ7|A0A2J6LNZ7_LACSA (437)	K01438	argE; acetylornithine deacetylase [EC:3.5.1.16]
tr|A0A2J6LI76|A0A2J6LI76_LACSA (623)	K02145	ATPeV1A, ATP6A; V-type H+-transporting ATPase subunit A [EC:7.1.2.2]
tr|A0A2J6LHJ7|A0A2J6LHJ7_LACSA (389)	K00218	por; protochlorophyllide reductase [EC:1.3.1.33]
tr|A0A2J6K7V0|A0A2J6K7V0_LACSA (477)		
tr|A0A2J6MJP6|A0A2J6MJP6_LACSA (635)	K08991	MUS81; crossover junction endonuclease MUS81 [EC:3.1.22.-]
tr|A0A2J6LDW8|A0A2J6LDW8_LACSA (249)	K02991	RP-S6e, RPS6; small subunit ribosomal protein S6e

tr|A0A2J6LC77|A0A2J6LC77_LACSA (1974)	K02349	POLQ; DNA polymerase theta [EC:2.7.7.7]
tr|A0A2J6LC09|A0A2J6LC09_LACSA (622)	K17681	ATAD3A_B; ATPase family AAA domain-containing protein 3A/B
tr|A0A2J6LBI7|A0A2J6LBI7_LACSA (404)	K01623	ALDO; fructose-bisphosphate aldolase, class I [EC:4.1.2.13]
tr|A0A2J6LBG3|A0A2J6LBG3_LACSA (894)	K03696	clpC; ATP-dependent Clp protease ATP-binding subunit ClpC
tr|A0A2J6JY99|A0A2J6JY99_LACSA (249)	K08909	LHCA3; light-harvesting complex I chlorophyll a/b binding protein 3
tr|A0A2J6JLK0|A0A2J6JLK0_LACSA (449)	K03231	EEF1A; elongation factor 1-alpha
tr|A0A2J6L776|A0A2J6L776_LACSA (511)	K18757	LARP1; la-related protein 1
tr|A0A2J6L5J6|A0A2J6L5J6_LACSA (1093)	K11838	USP7, UBP15; ubiquitin carboxyl-terminal hydrolase 7 [EC:3.4.19.12]
tr|A0A2J6L1L3|A0A2J6L1L3_LACSA (177)	K01602	rbcS, cbbS; ribulose-bisphosphate carboxylase small chain [EC:4.1.1.39]
tr|A0A2J6L042|A0A2J6L042_LACSA (479)		
tr|A0A2J6KTT7|A0A2J6KTT7_LACSA (552)	K02147	ATPeV1B, ATP6B; V-type H+-transporting ATPase subunit B
tr|A0A2J6MCD9|A0A2J6MCD9_LACSA (508)	K02111	ATPF1A, atpA; F-type H+/Na+-transporting ATPase subunit alpha [EC:7.1.2.2 7.2.2.1]
